# Factors associated with the use and quality of antenatal care in Nepal: a population-based study using the demographic and health survey data

**DOI:** 10.1186/1471-2393-14-94

**Published:** 2014-03-03

**Authors:** Chandni Joshi, Siranda Torvaldsen, Ray Hodgson, Andrew Hayen

**Affiliations:** 1 School of Public Health and Community Medicine, University of New South Wales, Sydney, Australia; 2 Australians for Women’s Health, Port Macquarie, NSW, Australia

**Keywords:** Prenatal care, Antenatal care, Prepartum care, Demographic and health survey, South Asia, Quantity of antenatal care, Timing of antenatal care

## Abstract

**Background:**

Good quality antenatal care (ANC) reduces maternal and neonatal mortality and improves health outcomes, particularly in low-income countries. Quality of ANC is measured by three dimensions: number of visits, timing of initiation of care and inclusion of all recommended components of care. Although some studies report on predictors of the first two indicators, no studies on the third indicator, which measures quality of ANC received, have been conducted in Nepal. Nepal follows the World Health Organization’s recommendations of initiation of ANC within the first four months of pregnancy and at least four ANC visits during the course of an uncomplicated pregnancy. This study aimed to identify factors associated with 1) attendance at four or more ANC visits and 2) receipt of good quality ANC.

**Methods:**

Data from Nepal Demographic and Health Survey 2011 were analysed for 4,079 mothers. Good quality ANC was defined as that which included all seven recommended components: blood pressure measurement; urine tests for detecting bacteriuria and proteinuria; blood tests for syphilis and anaemia; and provision of iron supplementation, intestinal parasite drugs, tetanus toxoid injections and health education.

**Results:**

Half the women had four or more ANC visits and 85% had at least one visit. Health education, iron supplementation, blood pressure measurement and tetanus toxoid were the more commonly received components of ANC. Older age, higher parity, and higher levels of education and household economic status of the women were predictors of both attendance at four or more visits and receipt of good quality ANC. Women who did not smoke, had a say in decision-making, whose husbands had higher levels of education and were involved in occupations other than agriculture were more likely to attend four or more visits. Other predictors of women’s receipt of good quality ANC were receiving their ANC from a skilled provider, in a hospital, living in an urban area and being exposed to general media.

**Conclusions:**

Continued efforts at improving access to quality ANC in Nepal are required. In the short term, less educated women from socioeconomically disadvantaged households require targeting. Long-term improvements require a focus on improving female education.

## Background

Worldwide, complications during pregnancy, childbirth and the postnatal period are the leading causes of death and disability among women of reproductive age [1]. In 2010, there were around 287,000 maternal deaths globally [2]. A large majority of these deaths are preventable [3] and almost all of the deaths (99%) occur in low-income countries [3]. Additionally, for every maternal death, an estimated 20 women suffer injury, infection or other morbidity [4]. Nepal has a high maternal mortality ratio at 281/100,000 live births [5].

Good quality antenatal care (ANC) can reduce maternal morbidity and mortality and perinatal mortality [3,6]. The quality of ANC is measured by three dimensions: number of visits, timing of initiation of care and inclusion of all recommended components of care [7]. Good quality ANC improves maternal health, decreases the chances of suffering from anaemia, pregnancy induced hypertension and preterm labour [8-10] and promotes positive pregnancy outcomes, including a reduced risk of low birth weight (<2,500 grams) and preterm babies [9-11]. ANC increases the use of a Skilled Birth Attendant (SBA; a doctor, nurse or midwife) during delivery and postnatal care possibly because the visit can be an opportunity to educate women about the merits of skilled birth attendance [12]. ANC visits provide an excellent opportunity to deliver education regarding the danger signs and symptoms during pregnancy, delivery and the postpartum period and to focus on birth spacing and family planning [13]. A study from Bangladesh found that women who had at most one ANC visit were twice as likely to suffer a perinatal death compared to women who had three or more ANC visits [14]. Early initiation of ANC and attendance at four or more ANC visits are associated with higher infant birth weights and lower infant mortality rates [15,16]. The timing of the first ANC visit, as well as the total number of ANC visits also affect the quality of ANC that a pregnant woman receives [17].

Nepal follows the World Health Organization’s recommendations of initiation of ANC within the first four months of pregnancy and at least four ANC visits during the course of an uncomplicated pregnancy [7]. Several studies from low-income countries, including a systematic review, depict a positive but weak association between the number of antenatal visits and maternal and child health outcomes such as maternal complications [18] and mortality [19], stillbirths [20] and low birth weight [18]. Such results have provided impetus for investigation into the components and quality of ANC [21,22]. In addition to the number of visits, the components included in ANC greatly influence its effectiveness and might also affect women’s decisions regarding the time of initiation and continuity of care [18,19]. Poor quality ANC has the potential to reduce its use [20].

The components of ANC suggested in Nepal include: iron supplementation, blood and urine tests, at least two tetanus toxoid injections, measurement of blood pressure, intestinal parasite drugs and health education regarding their pregnancy [23]. The different components of ANC improve maternal and child health in different ways [24,25]. Iron supplementation reduces the proportion of women becoming anaemic by increasing haemoglobin up to 0.7gram/decilitre per week; screening for hypertension and proteinuria allows early detection and treatment for preeclampsia and reduces case fatality of this condition; screening for infection reduces fetal loss and maternal and infant morbidity, preterm and low birth weight babies. Administration of antenatal tetanus vaccination virtually eliminates this condition in neonates [26].

Receiving ANC from an SBA, having at least four ANC visits which include blood pressure measurement, blood and urine tests and advice on pregnancy complications and where to go in case of such complications, have been shown to decrease the risk of neonatal mortality [27,28]. They have also been shown to increase rates of immunisation; enhance the chances of initiating breastfeeding within one hour of birth and the maintenance of exclusive breastfeeding for more than four months [27]; and increase the use of postnatal check-ups [29,30]. An Indian study found that, compared to women who had neonatal deaths, those with a live birth had received better quality ANC which included body weight measurement, blood and urine testing, a full course of iron tablets, tetanus toxoid injections, abdominal examination and ultrasonography [24].

The recommended number of visits is not always met in Nepal where more than a quarter (26%) of Nepalese women reported no ANC visits and only 29% reported four or more ANC visits [5]. Another Nepalese study (2013) showed that younger women, living in urban areas, having primary education or higher, with lower parity, from non-farming occupations, in higher wealth quintiles, who did not smoke and whose husbands also had primary education or higher, were more likely to attend four or more ANC visits and receive higher quality ANC [27].

There are few studies focusing on the quality of ANC in low-income countries including Nepal [31,32]. This study aimed to investigate factors associated with 1) four or more ANC visits and 2) receipt of good quality ANC, among Nepalese women who had given birth in the previous five years.

## Methods

### Data source

This study used data from the 2011 Nepal Demographic and Health Survey (DHS), a nationally representative household cluster survey of 10,826 households which interviewed 12,674 women aged 15–49 years and 4,121 men aged 15–49 years and had a response rate of 99% [33]. We restricted our analyses to the 4,079 women who gave birth in the five years preceding the survey. Among women with two or more live births in the five years preceding the survey, we referred to the most recent birth only. Full details of the survey have been published elsewhere [34].

In the Nepal DHS, all women who gave birth between 2007 and 2011 were asked a number of questions about antenatal health care. Information was collected about the person and institution providing ANC (if any), the number of ANC visits, the timing of the first ANC visit and the components included in the ANC provided. These components were: blood pressure measurement; urine testing for the detection of bacteriuria and proteinuria; blood tests for syphilis and anaemia; and the provision of iron supplementation, intestinal parasite drugs, tetanus toxoid injections and health education during pregnancy. Questions on health education were related to the provision of information on danger signs during pregnancy, where to go in case of such complications, and recommendations to use a skilled birth attendant for delivery [34].

### Outcome variables

1. We used ‘attendance at four or more ANC visits’ as our first outcome variable based on the World Health Organization recommendations on ANC [35].

2. We defined good quality ANC as that which included all seven recommended items of ANC in Nepal. These items are: the provision of iron supplementation, blood tests, urine tests, at least two tetanus toxoid injections, measurement of blood pressure, provision of intestinal parasite drugs and health education regarding their pregnancy [23]. We defined health education as the receipt of any one of the three items of health education during ANC: a recommendation to use a skilled birth attendant; education on the warning signs that may suggest significant pregnancy complications; or advice on where to seek health care, should problems develop.

### Study variables

Sixteen variables were considered for their potential association with attendance at four or more ANC visits. These were: women’s age at birth of the child, women’s education (no education, primary, secondary and tertiary education), women’s work status in the past 12 months (whether in paid employment at the time of the survey or the previous12 months), the quintile of wealth of women’s household (the DHS wealth quintile is a composite indicator which divides the households into five categories: poorest, poorer, middle, richer and richest; and were derived using principle component analysis based on information from housing characteristics and ownership of household durable goods) [36], religion (Hindu, Buddhist, Muslim, Kirat and Christian), smoking status of women, decision-making power (whether women participated in at least one of the decisions regarding their own health care, major household purchases or visits to their family or relatives), general media exposure (whether exposed to either radio, television or newspapers/magazines at least once a week), parity, whether the pregnancy was wanted at the time (the survey had three categories: ‘pregnancy wanted at that time’, ‘pregnancy wanted later’ and ‘pregnancy not wanted at all’ of which the later two were merged into one to form the response ‘No’ and the first one remained as ‘Yes’) and history of previous pregnancies (including history of miscarriages, stillbirths or neonatal deaths and multiple pregnancies), use of modern family planning method (the survey data had four categories: ‘No method’, ‘Folkloric method’, ‘Traditional method’ and ‘Modern method’; the first three categories were merged to form the response ‘No’ and the fourth category formed the response ‘Yes’), ecological zones (Mountains, Hills or Terai), residence (urban, rural), husbands’ education (no education, primary, secondary, tertiary and don’t know) and their occupation (agricultural, professional/technical/managerial, clerical, services, skilled manual, unskilled manual and other).

Twelve variables were considered for their potential association with receipt of good quality ANC: women’s age at birth of the child, women’s education, wealth quintile, decision-making power of women, general media exposure, parity, history of previous pregnancies, use of modern family planning methods, ecological zones, residence, place of receipt of ANC and the type of health worker providing ANC. The variable ‘place of receipt of ANC’ specified the highest place where the women got ANC and originally had 16 categories which were reduced to 9. The two responses ‘respondent’s home’ and ‘Other’s home’ were merged to form one category ‘Home’. The four main Non-Government Organisations (NGOs) providing services plus the ‘other NGO’ were merged into one response ‘NGO’. The two responses ‘Others’ and “Other Government’ were merged into one category ‘Other’. The two responses ‘Private Hospital/Clinic’ and ‘Other Private’ were merged into one category ‘Private Hospital/Clinic’. ‘Type of health worker’ had 8 categories reduced to six (Doctor, Female Community Health Volunteer, Village Health Worker, Maternal and Child Health Worker, Health Assistant/Auxiliary Health Worker and Nurse/Midwife). The two additional categories in the survey: ‘Traditional Birth Attendant’ and ‘Other’ were excluded as there were only five responses to each.

### Statistical analysis

All analyses used sampling weights and adjusted for the sampling design (clustering and stratification). We used linearization to obtain valid estimates of standard errors [37]. Firstly, we calculated descriptive statistics (frequencies or means and standard deviations as appropriate) and their 95% confidence intervals. We then calculated unadjusted odds ratios and 95% confidence intervals using logistic regression models to examine the association between the study factors and the receipt of: 1) four or more ANC visits; and 2) good quality ANC. For the variables mother’s age and parity, we checked their linearity using a lowess smoother [37]. The lowess smoother indicated a quadratic relationship between mother’s age and both outcomes, and so this variable was modelled using a quadratic term in both of the models. Because of the inclusion of a quadratic term, we centred mother’s age at its mean (25.4 years) to avoid problems with collinearity. We then fitted multivariable logistic regression models as follows:

We initially retained all variables that were significant at P < 0.25 in the univariable models. We then used a backwards elimination procedure, by removing the least significant variable in the model. This was repeated until all variables in the model had a P-value of < 0.05. For the model of quality of ANC, we retained the variable mother’s education in the final model as its significance was slightly above 0.05. In both models, we checked whether a quadratic term for mothers’ age was still required; in both cases, the model with a linear term only was adequate.

The analyses were undertaken in SAS 9.3 and Stata 12.1.

#### Ethics

The Nepal DHS surveys were approved by the Nepal Health Research Council, Kathmandu, Nepal and ICF Macro Institutional Review Board, Maryland, USA. Respondents provided written consent for the surveys [34]. We obtained permission to use this data from MEASURE DHS, which is the monitoring and evaluation body of the DHS globally. For this study, ethics approval was obtained from the Medical and Community Human Research Ethics Advisory Panel of the University of New South Wales (reference number 2012-7-28).

## Results

There were 4,079 women included in the analysis. After accounting for sample weights, this corresponded to 4,148 women. Table 1 provides characteristics of the women and their ANC.

**Table 1:** Factors associated with four or more antenatal care visits in Nepal

**Study variables**^**1**^	**Total number of women**	**Number (%) of women who had 4 or more ANC visits (%)**	**Unadjusted OR (95% CI)**	**P**	**Adjusted OR (95% CI)**	**P**
**Women’s age at birth of child in years (Mean, SD)**	25.3	24.3 (4.87)		<0.001		0.021
For every ten years increase in age of women	Linear		0.66 (0.57 to 0.77)	<0.001	1.34 (1.05 to 1.72)	
	Quadratic		0.63 (0.52 to 0.76)	<0.001		
**Women’s education**				<0.001		<0.001
No education	1822	523 (28.7)	1 (Referent category)		1 (referent category)	
Primary education	835	431 (51.6)	2.65 (2.15 to 3.26)		1.72 (1.38 to 2.14)	
Secondary education	1229	881 (71.7)	6.28 (5.00 to 7.89)		2.50 (1.94 to 3.22)	
Tertiary education	263	243 (92.6)	30.86 (15.67 to 60.77)		7.11 (3.28 to 15.44)	
**Women’s work status in the past 12 months**				0.001	-	-
Didn't work in paid employment	1150	652 (56.7)	1 (Referent category)			
Worked in paid employment	2999	1426 (47.5)	0.69 (0.56 to 0.86)			
**Wealth quintile to which the women’s household belonged**				<0.001		<0.001
Poorest	979	277 (28.3)	1 (Referent category)		1 (Referent category)	
Poorer	899	352 (39.1)	1.63 (1.26 to 2.11)		1.17 (0.88 to 1.56)	
Middle	873	419 (48.0)	2.35 (1.71 to 3.21)		1.28 (0.90 to 1.83)	
Richer	748	487 (65.1)	4.74 (3.44 to 6.54)		1.87 (1.28 to 2.74)	
Richest	649	543 (83.7)	13.03 (9.28 to 18.30)		3.00 (1.95 to 4.60)	
**Religion**				0.023	-	-
Hindu	3444	1787 (51.9)	1 (Referent category)			
Buddhist	360	158 (43.9)	0.73 (0.52 to 1.02)			
Muslim	235	82 (34.9)	0.50 (0.30 to 0.82)			
Kirat	58	29 (50.1)	0.93 (0.45 to 1.92)			
Christian	51	22 (43.4)	0.71 (0.37 to 1.36)			
**Women’s smoking status**				<0.001		0.036
Smokers	511	133 (26.1)	1 (Referent category)		1 (Referent category)	
Non-smokers	3638	1945 (53.5)	3.26 (2.43 to 4.38)		1.40 (1.02 to 1.92)	
**Whether women participated in at least one of the three decisions regarding their own health care, major household purchases and visit to their family or relatives**				0.005		0.005
No	2473	1134 (45.9)	1 (Referent category)		1 (Referent category)	
Yes	1675	943 (56.3)	1.52 (1.27 to 1.82)		1.32 (1.09 to 1.60)	
**Whether women were exposed to any of the three general media (radio, television or newspaper/magazines) at least once a week**				0.008	-	-
No	1641	754 (46.0)	1 (Referent category)			
Yes	2507	1324 (52.8)	1.32 (1.08 to 1.61)			
**Women’s parity (Mean, SD)**	2.6	2.1 (1.29)		<0.001		<.001
For each unit increase in live birth			0.65 (0.61 to 0.70)		0.78 (0.71 to 0.86)	
**Whether pregnancy wanted at the time**				0.001	-	-
Wanted pregnancy	3017	1582 (52.4)	1 (Referent category)			
Unwanted pregnancy	1131	496 (43.8)	0.71 (0.58 to 0.86)			
**History of previous pregnancies**				0.011	-	-
No complications in previous pregnancies	3484	1777 (51.0)	1 (Referent category)			
Complications in previous pregnancies	664	301 (45.3)	0.79 (0.67 to 0.95)			
**Use of modern family planning**				0.717	-	-
No	2781	1385 (49.8)	1 (Referent category)			
Yes	1367	693 (50.7)	1.04 (0.86 to 1.25)			
**Ecological zones of residence**				0.179	-	-
Terai	2174	1108 (50.9)	1 (Referent category)			
Hill	1669	840 (50.3)	0.98 (0.70 to 1.36)			
Mountain	306	130 (42.7)	0.72 (0.49 to 1.05)			
**Residence**				<0.001	-	-
Rural	3730	1778 (47.7)	1 (Referent category)			
Urban	418	300 (71.8)	2.79 (2.13 to 3.65)			
**Husband’s education**				<0.001		0.010
No education	872	200 (23.0)	1 (Referent category)		1 (Referent category)	
Primary	984	395 (40.2)	2.26 (1.67 to 3.04)		1.55 (1.12 to 2.13)	
Secondary	1809	1104 (61.1)	5.26 (3.94 to 7.01)		1.81 (1.31 to 2.51)	
Tertiary	461	367 (79.7)	13.15 (8.89 to 19.45)		1.60 (0.98 to 2.61)	
Don’t know	22	10 (45.0)	2.75 (0.84 to 8.95)		2.54 (0.74 to 8.74)	
**Husband’s occupation**				<0.001		0.002
Agricultural (self-employed & employee)	1005	334 (33.2)	1 (Referent category)		1 (Referent category)	
Professional/technical/managerial	210	173 (82.3)	9.34 (5.88 to 14.84)		2.13 (1.27 to 3.58)	
Clerical	487	265 (54.4)	2.40 (1.59 to 3.63)		1.44 (0.96 to 2.14)	
Services	991	661 (66.7)	4.02 (3.12 to 5.18)		1.76 (1.29 to 2.39)	
Skilled manual	680	312 (45.9)	1.70 (1.34 to 2.18)		1.36 (1.02 to 1.82)	
Unskilled manual	635	266 (42.0)	1.45 (1.08 to 1.96)		1.47 (1.05 to 2.05)	
Other	140	67 (47.4)	1.81 (0.97 to 3.37)		0.83 (0.42 to 1.63)	

### Number and quality of ANC

Half the women (n = 2078, 50.0%, 95% CI = 46.1 to 53.8%) had four or more ANC visits, whereas the other half had fewer than 4 ANC visits, including 15% of women who had no ANC at all. A total of 3,468 (3520 after weighting) women who had at least one ANC visit were included in the analysis for quality of ANC. A larger proportion of women attending four or more ANC visits received good quality ANC, compared to those who attended fewer than four ANC visits (84% vs 16%).

Three variables were highly significant (P < 0.001) for the association with four or more ANC visits in the multivariable model: women’s education, the wealth quintile to which their households belonged and their parity. With increasing education levels of women, their odds of receiving four or more ANC visits also increased, which was as high as seven times for women with tertiary education compared to those with no education (OR = 7.11; 95% CI: 3.28 to 15.44). Increasing wealth of women’s household also increased the odds of women getting four or more ANC visits, as women in the richest quintile had three times the odds of receiving four or more ANC visits than women in the poorest quintile (OR = 3.00; 95% CI: 1.95 to 4.60). Women of higher parity had lower odds of receiving four or more ANC visits. Women’s decision-making power (P = 0.005), and their husbands’ education (P = 0.010) and occupation (P = 0.002) were also significant in the model (Table 1). Women who participated in at least one household decision-making had higher odds of receiving four or more ANC compared to women who did not participate in any decision-making. The levels of husbands’ education increased the odds of women getting four or more ANC visits. Women whose husbands were involved in agriculture had much lower odds of having four or more visits compared to those involved in other occupations, including professional/technical/managerial jobs, clerical, skilled or unskilled manual jobs and services (Table 1).

Other variables significantly associated with four or more ANC visits in the same model were women’s age at birth of the child (P = 0.021) and their smoking status (P = 0.036). Non-smoking women had greater odds of attending four or more ANC visits, as did older women. However, variables including whether women were employed in the past 12 months, their religion, their general media exposure, whether the pregnancy was wanted, history of previous pregnancies, whether women resided in rural or urban area, which were all significant in the univariable model, lost their significance in the multivariable model (Table 1).

Among women who had at least one ANC visit, some components of ANC were more commonly received than others. Health education, iron supplementation, blood pressure measurement and two or more tetanus toxoid injections each were received by at least three quarters of the women while 64% were given intestinal parasite drugs, 56% had a urine sample taken and 45% had a blood sample taken (Table 2).

**Table 2:** Quality of antenatal care received during last pregnancy for Nepalese women

**N = 3520**	**Total number of women**	**%**	**95% CIs**
**Given information during pregnancy** (Told about pregnancy complications or told where to go for pregnancy complications or advised to use a skilled birth attendant)			
Yes	2,872	81.6	78.5 to 84.4
**Iron tablets/syrup taken**			
Yes	3,214	91.3	89.3 to 93.0
**Given intestinal parasite drugs**			
Yes	2,250	63.9	60.8 to 66.9
**Two or more tetanus injections given**			
Yes	3,033	86.2	84.2 to 87.9
**Blood pressure measured**			
Yes	3,043	86.4	83.9 to 88.6
**Blood sample taken**			
Yes	1,595	45.3	41.8 to 48.9
**Urine sample taken**			
Yes	1,968	55.9	52.4 to 59.3
**Received all seven ANC components**			
	854	24.3	21.6 to 27.2

Two variables were highly associated (P <0.001) with receipt of good quality ANC: the health worker who provided the care and the woman’s parity (Table 2). The type of health worker providing ANC was a very strong predictor for the receipt of good quality ANC. Compared to women who received ANC from a doctor, those who received ANC from a Village Health Worker had 96% lower odds of receiving good quality ANC (OR = 0.04; 95% CI: 0.01 to 0.29); those being served by a Female Community Health Volunteer had 90% lower odds (OR = 0.10; 95% CI: 0.01 to 0.62), those receiving service from a Maternal and Child Health Worker had 84% lower odds (OR = 0.16; 95% CI: 0.07 to 0.41), those receiving service from a Health Assistant or an Auxiliary Health Worker had 75% lower odds (OR = 0.25; 95% CI: 0.37 to 0.46) and those being served by a nurse had 26% lower odds (OR = 0.74; 95% CI: 0.58 to 0.94) of getting good quality ANC. With each unit increase in parity, the odds of receiving good quality ANC decreased by 21% (OR = 0.79; 95% CI: 0.70 to 0.88; Table 2).

Women’s age at birth of the child (P = 0.041), their education (P = 0.057), wealth quintile to which their households belonged (P = 0.001), their residence (P = 0.013), the place where ANC was received (P = 0.005) and modern family planning use by the women (P = 0.045) were all significant in the model. Older women received good quality ANC compared to younger women. Women with a tertiary education had one-and-half times greater odds of receiving good quality ANC compared to those with no education (OR = 1.53; 95% CI: 0.97 to 2.41). Women in the richest quintile had three times the odds of receiving good quality ANC compared to women in the poorest quintile (OR = 2.88; 95% CI: 1.70 to 4.89). Women residing in urban areas had greater odds of receiving good quality ANC compared to those living in the rural areas. Women using modern family planning had decreased odds of receiving good quality ANC (Table 3).

**Table 3:** Factors associated with quality of antenatal care services in Nepal

**Study variables**	**Total number of women**	**Number of women who had good quality ANC (%)**	**Unadjusted odds ratio (95% CI)**	**P**	**Adjusted odds ratio (95% CI)**	**P**
**Women’s age at birth of child** (Mean, SD)	24.7	24.2 (4.86)				0.041
With each ten years increase in women's age	Linear		0.82 (0.67 to 1.00)	0.045	1.46 (1.08 to 1.97)	0.013
	Quadratic		0.72 (0.58 to 0.90)	0.004		
**Women’s education**				<0.001		0.057
No education	1372	179 (13.1)	1 (Referent category)		1 (Referent category)	
Primary education	714	138 (19.3)	1.60 (1.16 to 2.20)		1.16 (0.81 to 1.68)	
Secondary education	1172	415 (35.4)	3.65 (2.64 to 5.05)		1.58 (1.09 to 2.28)	
Tertiary education	262	121 (46.2)	5.72 (3.79 to 8.62)		1.53 (0.97 to 2.41)	
**Wealth quintile to which the women’s household belonged**				<0.001		0.001
Poorest	657	55 (8.3)	1 (Referent category)		1 (Referent category)	
Poorer	733	98 (13.3)	1.69 (1.11 to 2.57)		1.40 (0.90 to 2.19)	
Middle	792	169 (21.3)	2.98 (1.95 to 4.56)		1.90 (1.18 to 3.06)	
Richer	701	256 (36.6)	6.34 (4.12 to 9.73)		2.86 (1.74 to 4.71)	
Richest	637	276 (43.3)	8.40 (5.44 to 12.98)		2.88 (1.70 to 4.89)	
**Whether women participated in at least one of the three decisions regarding their own health care, major household purchases and visit to their family or relatives**				0.043	-	-
No	2059	467 (22.7)	1 (Referent category)			
Yes	1461	386 (26.4)	1.22 (1.01 to 1.49)			
**Whether women were exposed to any of the three general media (radio, television or newspaper/magazines) at least once a week**				0.329	-	-
Not exposed	1334	340 (14.6)	1 (Referent category)			
Exposed	2186	514 (9.7)	0.90 (0.72 to 1.12)			
**Parity (Mean, SD)**	2.4	1.9 (2.00)		<0.001		<.001
With each unit increase in live birth			0.69 (0.63 to 0.75)		0.79 (0.70 to 0.88)	
**History of previous pregnancies**				0.016	-	-
No complications in previous pregnancies	2978	750 (25.2)	1 (Referent category)			
Complications in previous pregnancies	542	104 (19.2)	0.71 (0.53 to 0.94)			
**Use of modern family planning**				0.044		0.045
No	2337	595 (25.5)	1 (Referent category)		1 (Referent category)	
Yes	1183	258 (21,8)	0.82 (0.67 to 0.99)		0.79 (0.62 to 0.99)	
**Ecological zones where women resided**				0.001	-	-
Terai	1956	551 (28.2)	1 (Referent category)			
Hill	1328	262 (19.7)	0.63 (0.45 to 0.87)			
Mountain	237	41 (17.3)	0.53 (0.36 to 0.78)			
**Residence**				<0.001		0.013
Rural	3128	722 (23.1)	1 (Referent category)			
Urban	392	132 (33.7)	1.69 (1.29 to 2.20)		1.52 (1.09 to .2.12)	
**Place where women received antenatal care**				<0.001		0.005
Government Hospital	605	210 (34.7)	1 (Referent category)		1 (Referent category)	
Home	43	1 (2.8)	0.05 (0.01 to 0.39)		0.25 (0.03 to 2.08)	
Government PHC Centre	287	79 (27.6)	0.72 (0.46 to 1.11)		0.85 (0.52 to 1.37)	
Government Health Post	430	54 (12.6)	0.27 (0.18 to 0.41)		0.44 (0.28 to 0.68)	
Government Sub Health Post	877	88 (10.0)	0.21 (0.14 to 0.32)		0.67 (0.40 to 1.11)	
Government PHC Outreach	269	30 (11.1)	0.23 (0.11 to 0.48)		0.94 (0.50 to 1.77)	
Private Hospital/Clinic	860	322 (37.4)	1.12 (0.84 to 1.51)		0.85 (0.62 to 1.17)	
NGO	136	68 (49.8)	1.86 (1.15 to 3.01)		1.60 (1.02 to 2.52)	
Others	13	2 (12.7)	0.27 (0.07 to 1.03)		0.55 (0.10 to 3.07)	
**Health worker who provided the women with antenatal care**				<0.001		<0.001
Doctor	1115	462 (41.5)	1 (Referent category)		1 (Referent category)	
Female Community Health Volunteer	38	1 (1.5)	0.02 (0.00 to 0.10)		0.10 (0.01 to 0.62)	
Village Health Worker	56	1 (1.5)	0.02 (0.00 to 0.16)		0.04 (0.01 to 0.29)	
Maternal and Child Health Worker	534	25 (4.7)	0.07 (0.03 to 0.16)		0.16 (0.07 to 0.41)	
Health Assistant/Auxilliary Health Worker	475	35 (7.5)	0.11 (0.07 to 0.20)		0.25 (0.13 to 0.46)	
Nurse/Midwife	1301	329 (25.3)	0.48 (0.39 to 0.59)		0.74 (0.58 to 0.94)	

The place where ANC was provided was associated with receipt of good quality ANC. Compared to those receiving ANC from a government hospital, those who visited a Non-Government Organisation had more than one-and-a-half times greater odds of receiving all seven ANC items (OR = 1.60; 95% CI: 1.02 to 2.52). However, women who received care at any other places [including Government Primary Health Care (PHC) outreach, Government Sub-Health Post, Government Health Post, Government PHC, private hospital/clinic and home] had lower odds of receiving good quality ANC compared to those who received ANC at a government hospital.

On the other hand, the decision-making power of women, the ecological zones where they lived and history of previous pregnancy lost their significance in the multivariable model (Table 3).

## Discussion

This study found that half of the Nepalese women who had given birth during the five year study period (2007 to 2011), had four or more ANC visits compared with only 29% during the previous survey [5]. However, fewer than a quarter of these women (24%) received good quality ANC. We found that receipt of four or more ANC visits and the quality of ANC were associated with a number of factors ranging from socio-economic to demographic factors and women’s empowerment.

### Four or more ANC visits

Women with higher levels of educational attainment were more likely to have four or more ANC visits. A study in Nepal on women’s empowerment and maternal health utilisation, analysing the 2001 DHS, also found that women's education was strongly associated with greater use of maternal health care [38]. Female education improves wealth, reduces gender disparity and empowers women [39]. Previous studies in South Asia suggest that education fosters new values and attitudes that are favourable to the use of modern health care, increases the chances of women desiring skilled care and empowers them to access such care [38]. Therefore, policies aimed at improving female education are likely to also improve ANC use. This is particularly important in Nepal, where only a very small proportion of Nepalese girls (6%) complete secondary school [35], only 18% of Nepalese women have a secondary education and the adult female literacy rate is 47% [40].

Educational levels of the husbands also showed a similar effect on the receipt of four or more ANC visits. This is similar to the results of a systematic review undertaken in low-income countries [41]. A randomised controlled trial on ANC in urban Nepal found that educating women and their husbands together resulted in women retaining more maternal health knowledge than if they attended alone, concluding that educational strategies become more effective when men are incorporated [42].

Women from higher income households had better odds of attending four or more ANC visits. This is similar to another study analysing data from 2006 Nepal DHS which found that women from higher wealth index household used ANC earlier during pregnancy and more frequently than those from lower wealth index households [29]. Women whose husbands were involved in agriculture had lower odds of four or more ANC visits than those whose husbands were involved in other occupations. In a study in rural Nepal, women whose husbands were involved in farming were less likely to have ANC than those who had any other jobs, linking farming with poverty and lack of disposable income [43].

The finding from this study that parous women have decreased odds of attending four or more ANC visits is consistent with findings from similar studies [41,44,45]. The second or later pregnancy carries lower risks for complications if the first pregnancy and birth were uncomplicated [33]. The experience of a previous pregnancy without complications might influence the perception of women about the need to seek care during pregnancy [46]. Some women might be occupied with the responsibilities of other children and hence find it difficult to attend ANC [41,46].

In this study, non-smoking women had higher odds of four or more ANC visits. A similar conclusion was drawn in Brazil [45]. Previous studies have shown use of fewer health care services in general and ANC in particular among smokers [35]. Nonetheless, a previous study in Nepal depicted no association between women’s smoking status and use of ANC when controlled for other socio-demographic factors [29].

Women who participated in at least one household decision-making factor had higher odds of receiving four or more ANC visits compared to women who had no decision-making participation. This is consistent with the findings of a previous study in Nepal in which women who had a say in decision-making regarding large household purchases had significantly higher chances of receiving ANC than those who did not [38].

### Quality of ANC

The type of health worker providing ANC plays a vital role in determining the quality of ANC received by women [19]. This study highlights the importance of skilled providers (doctors, nurses or midwives) in the provision of ANC. The odds of receiving good quality ANC were higher for women attending skilled providers compared with those who were relatively less skilled (Female Community Health Volunteers, Village Health Workers, Maternal and Child Health Worker and Health Assistant/Auxiliary Health Worker).

Women with higher levels of education were more likely to receive good quality care. A Ugandan study demonstrated the importance of women’s education for the receipt of all the recommended ANC components [31]. Women from higher income households had higher odds of receiving good quality ANC. This is consistent with studies from India and Nigeria which showed that higher socioeconomic status was associated with higher chances of using all ANC components [31,46,47]. More affordability and access to health information among women of higher socioeconomic status compared to women from lower socioeconomic status may explain these associations [29].

This study shows that the rurality of the place where women reside impacts on the quality of ANC. Women in urban areas had higher odds of receiving good quality ANC compared to those in the rural areas. This may be due to the fact that the rural areas have significantly less or even absent transportation infrastructure, making access to health care difficult [48].

Various ANC interventions can contribute to improving maternal and neonatal health. The majority of women in this study had their blood pressure measured, allowing for the detection of hypertension, which may represent an early sign of pre-eclampsia [35]. However, only just over half of the women had their urine tested for protein and infection, and even fewer (45%) had their blood taken to test for anaemia. These findings are comparable to a previous study which related such low utilisation with the lack of capacity of the health centres to carry out such tests [49]. Ninety-one per cent of women received iron supplements. The World Health Organization recommends providing iron supplementation to all pregnant women in developing countries at high risk of iron deficiency [13]. The proportion of women receiving tetanus toxoid injections, intestinal parasite drugs, and health education during pregnancy are similar to those from a Zambian study that used the 2007 DHS, but much higher than those from a Ugandan study [19,31].

#### Strengths and limitation of the study

This study has a number of strengths. The survey was a population-based study with a large sample size which was nationally representative. It was conducted rigorously using standard protocols and trained data collectors. The questionnaires were translated into three local languages and pretested. The response rate for the interviews was very high (99%).

Nonetheless, the survey had some limitations. The data were self-reported and it was a retrospective study making the information thus collected subject to recall bias. However, an effort was made to reduce the recall bias by analysing data on the most recent pregnancy within five years of the survey. As the data were reported by the women, there is no information on whether the healthcare workers provided the interventions in the optimal way.

The variable ‘Place of receipt of ANC’ may not fully capture the health facilities where women received ANC since only one response was allowed for this question, even though women may have received ANC at more than one type of health facility. Similarly, the variable ‘Health Worker providing ANC’ also only allowed one response, meaning that the type of health worker used in the analysis was not necessarily the worker women saw exclusively. Most of the variables (the smoking status of women, their education, occupation, parity, obstetric history, decision-making power, exposure to general media, wealth index and husband’s education and occupation) were measured at the time of the survey rather than at the time of birth of the child. These variables may have changed since the women gave birth. Finally, while women were required to receive at least two tetanus toxoid injections during pregnancy to meet the recommendation for protection against tetanus, we were unable to account fully for all tetanus toxoid vaccines taken prior to the most recent pregnancy. This could have affected the number of injections needed during the most recent pregnancy, and also meant that we may have underestimated the proportion of women who received all items of ANC.

## Conclusion

Only 50% of Nepalese women attended the recommended minimum of four antenatal visits during their last pregnancy. Given that only 16% of women who had fewer than four ANC visits received good quality ANC, encouraging women to attend at least four ANC visits must remain a priority, as must access to skilled providers of ANC. This study highlights the socio-economic factors associated with access to good quality ANC. It supports the targeting of less educated and socioeconomically disadvantaged women for participation in ANC as a short-term measure to improve outcomes. However, for longer term improvements in women’s access to good quality ANC, there needs to be a focus on improving the education of girls and women in Nepal as part of a strategy to improve gender equity and the empowerment of women.

## Abbreviations

ANC: Antenatal care; DHS: Demographic and health survey; NGOs: Non-government organisations; PHC: Primary health care; SBA: Skilled birth attendant.

## Competing interests

The authors declare that they have no competing interests.

## Authors’ contributions

CJ designed the study, worked on the analysis and drafted the manuscript. AH helped revise the study design, supervised the data analysis and conducted a part of it, drafted and revised the manuscript. ST helped revise the study design and drafting and revising the manuscript, and contributed to interpretation of the analysis. RH participated in the interpretation of the data, as well as revised the manuscript. All authors read and approved the final manuscript.

## Pre-publication history

The pre-publication history for this paper can be accessed here:

http://www.biomedcentral.com/1471-2393/14/94/prepub
